# Perspective Insight into Future Potential Fusion Gene Transcript Biomarker Candidates in Breast Cancer

**DOI:** 10.3390/ijms19020502

**Published:** 2018-02-07

**Authors:** Ryong Nam Kim, Hyeong-Gon Moon, Wonshik Han, Dong-Young Noh

**Affiliations:** 1 Center for Medical Innovation, Biomedical Research Institute, Seoul National University Hospital, Seoul 03080, Korea; 2 Department of Surgery, Seoul National University College of Medicine, Seoul 03080, Korea; moonhgsurg@gmail.com (H.-G.M.); han@snu.ac.kr (W.H.); dyno@snu.ac.kr (D.-Y.N.); 3 Cancer Research Institute, Seoul National University, Seoul 03080, Korea

**Keywords:** fusion gene transcript, breast cancer, biomarker

## Abstract

Next generation sequencing has accelerated the discovery of a variety of new fusion gene types in clinical breast cancer samples by analyzing cancer genomes and transcriptomes. Although previous studies have focused on a few clinically validated oncogenic fusion genes as diagnostic and therapeutic targets in breast cancer, a perspective consideration has not been given thus far for a plethora of breast cancer fusion genes, which are being newly identified at an overwhelmingly increasing pace. In this perspective review, we discuss diverse fusion gene types recently identified in a variety of breast cancer subtypes, including breast clinical cancer samples in TCGA (The Cancer Genome Atlas) database. This perspective review will confer fresh and promising guidance onto breast cancer surgeons, clinical oncologists, and tumor biologists in determining research directions for seeking and developing novel fusion gene biomarkers for breast cancer diagnostics and therapeutic treatment in upcoming years.

## 1. Introduction

Genomic rearrangements are well known to be closely associated with carcinogenesis in diverse types of human tissues [1,2,3]. Genomic rearrangements as diagnostic and therapeutic targets were initially identified in hematological malignancies, such as chronic myelogenous leukemia (CML) [4,5] and Burkitt’s lymphoma [6]. Recurrent translocations were subsequently reported in Ewing’s sarcoma [7,8] and synovial sarcoma [9]. Even though diverse kinds of genomic rearrangements, including conjoined genes (read-through fusion genes) [1], were previously known to be associated with carcinogenesis, a majority of the validated oncogenic fusion genes seem to be generated from the inter-chromosomal and intra-chromosomal rearrangements, thus far. With the advent of high-throughput next generation sequencing technology, substantial discovery of genomic rearrangements is now being accelerated at an unprecedented pace in diverse kinds of tumor types, including breast cancer. For instance, whole genome and whole exome sequencing and RNA-Seq recently enabled discoveries of *KIAA1217-RET* [2], *SEC31A-ALK* [3], *TPR-ALK* [4] and *HIP1-ALK* [5], all of which are chromosomal translocation-derived novel fusion oncogenes causing lung adenocarcinoma. Such genomic alterations have turned out to be diagnostic and therapeutic targets for treating lung cancer patients. 

In contrast to other cancer types, genomic rearrangements leading to the formation of fusion genes in breast cancer are, so far, less well known. In this perspective paper, we discuss fusion genes recently identified in breast cancer patients, including TCGA (The Cancer Genome Atlas) clinical breast cancer samples, addressing what kinds of fusion genes might be potential targets to prioritize in seeking future fusion gene biomarkers in breast cancer. 

## 2. Abundance of Diverse Kinds of Fusion Gene Types in Breast Cancer

**Figure 1:**
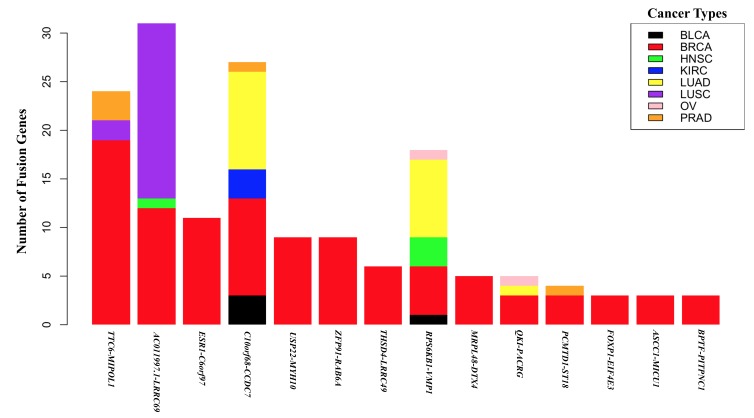
Occurrence of recurrent breast cancer transcript fusion genes across diverse cancer types. The stacked bar plot shows occurrence frequencies of the top 14 most recurrent breast cancer transcript fusion genes in 8 cancer types, BLCA (Bladder Urothelial Carcinoma), BRCA (breast Invasive Carcinoma), HNSC (Head and Neck Squamous Cell Carcinoma), KIRC (Kidney Renal Clear Cell Carcinoma), LUAD (Lung Adenocarcinoma), LUSC (Lung Squamous Cell Carcinoma), OV (Ovarian Serous Cystadenocarcinoma), and PRAD (Prostate Adenocarcinoma).

Recent study has shown us an overview of how many fusion genes there are across diverse cancer types in TCGA clinical cancer samples and what kinds of fusion types they are [6]. Fusion gene transcripts showing high recurrence in the breast invasive carcinoma samples are as follows: *TTC6-MIPOL1*, *AC011997.1-LRRC69*, *ESR1-C6orf97*, *C10orf68-CCDC7*, *USP22-MYH10*, *ZFP91-RAB6A*, *THSD4-LRRC49*, *RPS6KB1-VMP1*, *MRPL48-DTX4*, *QKI-PACRG*, *PCMTD1-ST18*, *FOXP1-EIF4E3*, *ASCC1-MICU1*, and *BPTF-PITPNC1*. Among the 14 fusion genes recurring in breast invasive carcinoma samples, *AC011997.1-LRRC69*, *C10orf68-CCDC7* and *RPS6KB1-VMP1* showed relatively high recurrence not only in breast carcinoma but also in LUSC (Lung squamous cell carcinoma) and in LUAD (lung adenocarcinoma) (Figure 1). The fusion genes in TCGA clinical breast cancer samples diverge greatly from one another in terms of translocation patterns, genomic positions conjoining between upstream and downstream partner genes, and functional classification of those partner genes. Nevertheless, no matter what kinds of genomic translocation might occur in generating such fusion genes, the prerequisite for creating oncogenic translocation and fusion gene transcripts should primarily be in-frame fusion and then the acquisition of optimal oncogenic function in those fusion proteins. Because this perspective review focuses mainly on fusion gene transcripts based on RNA-Seq data analysis, detailed explanations of genomic rearrangements are beyond the scope of our review, and could be found somewhere else.

If highly recurrent genomic translocation regions can be associated with frequent fusion events involving oncogenes, we should not neglect the high likelihood of as yet unidentified in-frame fusion events involving the same oncogenes in the genomic regions, and we should consider the genomic regions as target regions for screening oncogenic fusion genes. Recently, several pioneering studies have elucidated the reasonable cause of specific regions in the cancer genomes being more frequently broken and more recurrently involved in translocations than other regions, and how the epigenomic and genomic structural landscapes could affect the genomic structural fragility of these regions [7]. Notably, they found a new explanation in epigenetic aspects regarding such frequent structural fragility at specific genomic regions. The authors demonstrated that histone methylation modification mark H3K4me3 could be closely associated with genomic regions which are very vulnerable to DNA double strand breakage. 

**Figure 2:**
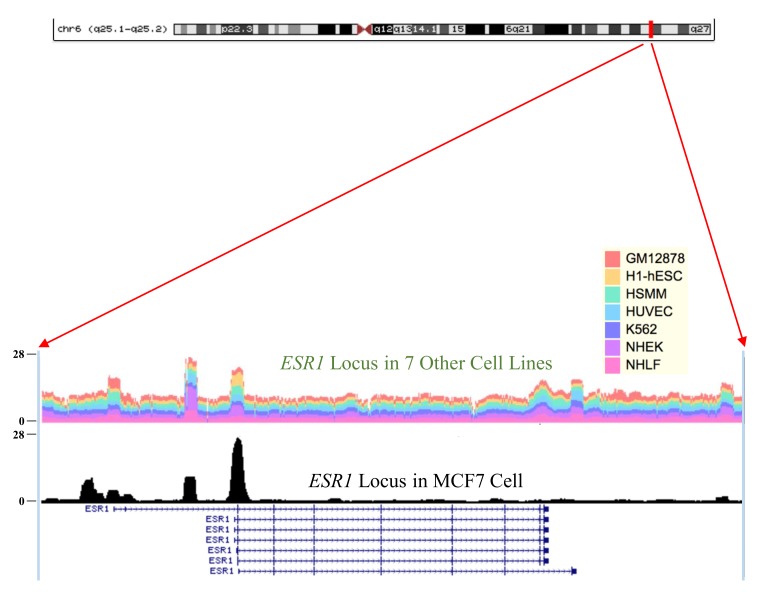
Strong H3K4me3 epigenetic modification signals within the *ESR1* locus in human breast adenocarcinoma MCF-7 cells. H3K4me3 signals in the MCF-7 *ESR1* locus are compared with the stacked spectra of those signals in the seven other cell lines, GM12878 (human lymphoblastoid cell), H1-hESC (H1-human Embryonic Stem Cell), HSMM (Human Skeletal Muscle Myoblast cell), HUVEC (Human Umbilical Vein Endothelial Cell), K562 (human immortalised myelogenous leukemia cell), NHEK (Normal Human Epidermal Keratinocytes), and NHLF (normal human lung fibroblasts). The *y*-axis indicates the intensity of the epigenetic modification H3K4me3 at each genomic position.

**Figure 3:**
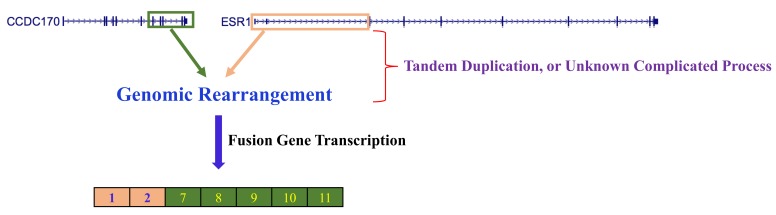
*ESR1-CCDC170* fusion gene transcript formation. The intron 2 region within the *ESR1* genomic locus appeared to undergo a DNA breakage, subsequently conjoining with the intron 6 region within the genomic locus of its neighboring gene *CCDC170*. This genomic rearrangement results in an oncogenic fusion mRNA transcript involving an upstream part (exons 1 and 2) of *ESR1* and the C-terminal portion (exons 7–11) of its neighboring gene *CCDC170*.

In order to check whether their assertion may also be applicable to the genomes in human breast adenocarcinoma cell line MCF-7, we analyzed ChIP-Seq dataset (GEO accession: GSE35583 [8]) for a genome-wide H3K4me3 landscape in MCF-7 cells. Intriguingly, we found that, in a genomic region harboring the exon 3 and its adjacent intron 2 portion within the *ESR1* gene locus, there are the two strongest histone-modified mark peaks for H3K4me3 in MCF-7 cells (Figure 2). More surprisingly, we found that the genomic region with the two strongest H3K4me3 marks is the region most vulnerable to DNA strand breakage; releasing a 5′ portion of *ESR1* which has exon 1 and exon 2 intact, which can often conjoin to the intron 6 region of the neighboring gene *CCDC170*, consequently causes the *ESR1-CCDC170* fusion gene (Figure 3) [9]. This finding corroborates their assertion that epigenetic mark H3K4me3 could be associated with genomic DNA breakage and subsequently with genomic rearrangements. In contrast to those epigenetic marks in MCF-7 cell, other human cells (GM12878, H1-hESC, HSMM, HUVEC, K562, NHEK and NHLF) did not exhibit such strong H3K4me3 marks in the genomic regions, explaining why *ESR1* could be involved in various fusion gene types only in breast carcinoma, but not in other human cell types (Figure 2). 

Such novel insight based on epigenomics may provide molecular pathologists and clinical tumor biologists with a fascinating and exciting clue for further delving deeper into the molecular mechanism of fusion gene formation in breast cancer and for discovering fusion gene biomarkers in the future. 

## 3. Recurrent in-Frame Fusion Genes across TCGA Clinical Breast Tumor Samples

There were 319 fusion genes in TCGA clinical breast cancer tumors checked in the previous study [6]. Among them, there were 16 recurrent in-frame fusion genes (*TTC6-MIPOL1*, *MRPL48-DTX4*, *THSD4-LRRC49*, *ASCC1-MICU1*, *QKI-PACRG*, *RPS6KB1-VMP1*, *RFTN1-DAZL*, *AFAP1-ABLIM2*, *SBNO2-C19orf20*, *MVP-KIF22*, *GIGYF2-EFHD1*, *ARFGEF1-CPA6*, *EFHD1-EIF4E2*, *SLC39A11-SDK2*, *BPTF-PITPNC1*, and *TBC1D4-COMMD6*) with frequency ≥2. Interestingly, of the 16 recurrent in-frame fusion genes, 13 (*MRPL48-DTX4*, *THSD4-LRRC49*, *ASCC1-MICU1*, *QKI-PACRG*, *AFAP1-ABLIM2*, *SBNO2-C19orf20*, *MVP-KIF22*, *GIGYF2-EFHD1*, *ARFGEF1-CPA6*, *EFHD1-EIF4E2*, *SLC39A11-SDK2*, *BPTF-PITPNC1*, and *TBC1D4-COMMD6*) had occurred only in breast cancer samples, but not in other cancer types. These in-frame fusion genes might be suitable candidate targets for screening fusion genes causing breast carcinogenesis in the future. In addition, the in-frame fusion genes generated through a joining between non-coding regions, like the breast cancer-specific oncogenic fusion gene *ESR1-CCDC170*, should be carefully identified by using whole genome sequencing and RNA-Seq data in the future. 

## 4. Fusion Genes Involving Tumor Suppressor Genes in Breast Cancer

Fusion genes involving tumor suppressor genes as fused partners that have been identified in the breast cancer samples are as follows: *TSC2-TMEM204*, *ARID1A-THBS3*, *ARID1A-YTHDF2*, *AXIN1-ITFG3*, *AXIN1-ITCH*, *AXIN1-IFT140*, *CASP8-ALS2CR12*, *CDH1-CCDC132*, *CDH1-NPFFR2*, *CDH1-DEXI*, *CCDC132-CDH1*, *ASXL1-TM9SF4*, *ASXL1-PDRG1* (Tumor suppressor genes are underlined in the names of fusion genes for clarity) (Table 1). Previous investigations had reported that *TSC2*, *ARID1A*, *AXIN1*, *CASP8*, *CDH1*, and *ASXL1* could play roles in tumor suppression in diverse cancer types, including breast cancer. Intriguingly, we found that the above-mentioned fusion events could break the functional domains of the tumor suppressor genes as the fusion partners, via the involvement of their incomplete functional domain structures in the fusion gene bodies, no matter whether the fusion styles are in-frame or out-of-frame (Table 1). This phenomenon might be another mechanism for causing the inactivity of tumor suppressor proteins, contributing to carcinogenesis of breast cells.

## 5. Fusion Genes Involving Kinase Partner Genes in Breast Cancer

Among 1019 TCGA breast clinical cancer samples tested in the previous study, 94 (9.2%) harbored in-frame fusion genes conjoined involving protein kinase partner genes. A total of 105 in-frame kinase fusion genes were identified in the breast cancer samples and, of them, the druggable kinase family partner genes involved in those in-frame fusions are *RET*, *NTRK3*, *FGFR1* and *FGFR2*. The kinase gene partners involved in those in-frame fusion genes belong to the following kinase gene families: CAMK Ser/Thr protein kinase family (*OBSCN-IGSF21*, *PHF21A-CAMK1D*, *TRIO-MARCH11*, *PIM3-CRELD2* and *SPG11-CAMK2G*), STE Ser/Thr protein kinase family (*TAOK1-ACER3* and *POLA1-MAP3K15*), AGC Ser/Thr protein kinase family (*CDC42BPB-KIAA1409*, *ADRBK2-SF3A1*, *PRKCA-CRYBA1*, *MAST4-NLN*, *MAST2-NASP*, *ROCK1-LRIG3*, *RPS6KA6-HDX*, *PRKCA-CDH8*, *RPS6KB2-PAAF1*, *PKN2-COL24A1*, *ADRBK1-BAIAP2* and *ADRBK1-IQGAP1*), CMGC Ser/Thr protein kinase family (*KHDC1-NLK*, *AHR-CDK14*, *PPP2CA-CDKL3*, *CDK12-FBXL20* and *MAPK14-EFHA1*), PI3/PI4-kinase family (*ATR-DBR1*, *SMG1-ANO3* and *PRKDC-CEP290*), Tyr protein kinase family (*TMEM87B-MERTK*, *SOX13-MERTK* and *ETV6-NTRK3*), TKL Ser/Thr protein kinase family (*KSR2-P2RX4*, *LRRK1-ADAMTS17*, *LIMK2-RUFY1* and *TESK2-GPR37L1*), ADCK protein kinase family (*ADCK2-DPP6*), and NEK Ser/Thr protein kinase family (*NVL-NEK2*). In contrast to most known kinase fusion cases, in which only one partner is a kinase gene, there is another kinase fusion (*ERBB2-CDK12*), in which both partners are kinase genes. The genomic regions easily vulnerable to the recurrent translocation breaks which generate those kinase fusion genes should be checked with priority given to screening potential candidate kinase oncogenic fusion targets in the coming years.

## 6. Fusions Involving Chromatin Modifier Genes in Breast Cancer 

Among 1019 breast cancer samples tested by Yoshihara et al. in their study [6], 38 (3.7%) harbored in-frame fusion involving chromatin modifier genes. Of those chromatin modifier genes involved in the fusion genes, there are genes encoding histone demethylase (*KDM5A-ANO2*, *USP21-PVRL4*, *WNK1-KDM5A*, *KDM5A-NINJ2*, *KDM4B-UHRF1*, *MYSM1-APOM*, and *KDM2A-RHOD*), NURD complex components (*CHD4-DTX1*, *XPC-RCOR1*, and *TRAF3-RCOR1*), histone methyltransferases (*NSD1-CDH23*, *CTPS2-RBBP7*, *CDH23-NSD1*, *MLL3-CLIP2*, *B4GALT7-NSD1*, *NSD1-ZNF346*, *SETDB1-PKP4*, and *SCMH1-EIF2B3*), SWI/SNF family members (*SMARCA4-DNM2*, *TBC1D9-SMARCA5*, *ARID1A-THBS3*, and *ARID1A-YTHDF2*), methyl-CpG-binding proteins (*MBD2-DOCK2*), histone acetyltransferases *(CREBBP-NUBP1*, *ARHGEF17-EP300*, and *CREBBP-ZNF500*), histone deacetylases (*HDAC1-KPNA6*, and *HDAC2-TRDN*), methylcytosine hydroxylase (*TET3-DGUOK*, and *TET3-C3ORF78*), 5_3_ORF chromatin modifier (*MANF-SETD2*) and histone demethylase_5_3_ORF chromatin modifier (*USP22-MYO19*) (Table 2).

An interesting thing to be mentioned here is the fusion event between *ARHGEF17* and *EP300*. *EP300*—a histone acetyltransferase involved in the regulation of transcription via chromatin remodeling—has recently been revealed as a tumor suppressor modulating paclitaxel resistance and stemness against metastatic breast cancer [10]. Intriguingly, by analyzing protein domains of the *EP300* region involved in the fusion event, we have found that, by omitting important functional domains (zf-TAZ and KIX) of *EP300*, the fusion *ARHGEF17-EP300* might cause a deficiency of *EP300* protein function, likely contributing to breast carcinogenesis and metastasis (Table 2).

Given the fact that epigenomic changes could exert significantly influential roles in malignant transformation, those fusion genes involving chromatin modifier partners should be good candidate targets for screening tumor suppressor or oncogenic fusion genes associated with breast carcinogenesis in the future.

## 7. Conjoined Genes in Breast Cancer

Conjoined genes, also called read-through fusion genes, have been reported in normal breast and breast cancer tissues by Kim et al. [1]. The authors discovered that those conjoined genes could be formed by a continuous read-through transcription from the upstream partner gene to the downstream partner gene due to a lack of poly-A signal sequence for stopping a transcription in the 3′UTR of the upstream partner gene. They also identified that the conjoined transcripts could exist in a variety of alternative splicing isoforms, among which some could have novel exons that could be formed by new exonization of certain lengths of sequences in the intergenic regions between the upstream and downstream fusion partner genes. Intriguingly, each isoform of the conjoined gene transcripts appeared to show a different tendency in expression level between breast cancer and normal tissues, suggesting that those conjoined gene transcript isoforms might play different roles between breast cancer and normal cells. Moreover, the authors showed that those conjoined genes could have been evolutionarily conserved between human and chimpanzee, suggesting that such conjoined gene formation could occur across a variety of species.

Similarly, Varley et al. found two recurrent read-through fusion transcripts, *SCNN1A-TNFRSF1A* and *CTSD-IFITM10,* that were present in breast cancer cell lines and estrogen receptor positive (ER+) breast primary tumor tissues, but not in normal breast tissues [11]. Both read-through fusion transcripts had in-frame open reading frames, whose translated proteins had been confirmed through Western blotting by the authors. They also demonstrated that breast cancer cell proliferation could be reduced by knock-downing *CTSD-IFITM10* mRNA expression via targeting its fusion junction region with custom small interfering RNAs, suggesting that the conjoined genes might, in the future, be a diagnostic biomarker and therapeutic target for treating a breast cancer subtype harboring those fusion biomarkers. 

An important thing to be addressed here is that not only those conjoined gene transcripts, but also numerous other conjoined gene transcripts could be formed through the read-through transcription between upstream and downstream genes, primarily depending upon the distance between them [12]. There may be numerous ways of forming fusions between neighboring genes, as shown in the fusion between the *ESR1* gene and its neighboring gene *CCDC170* by a tandem duplication [9]. Based upon this phenomenon, it is estimated that one third of all genes in the human genome might be involved in the formation of such conjoined genes with neighboring partner genes, even though the detection of all such conjoined genes is still difficult due to their extremely low expression level [12]. This fact implies that carcinogenesis might even be a far more complicated phenomenon than we imagined at present if a significant portion of such conjoined genes might be involved in the carcinogenesis. Understanding molecular mechanisms of how such conjoined genes could be implicated in the carcinogenesis may be an interesting research field in future years.

## 8. Fusion Gene Formation within Recurrent Genomic Copy Number Amplicon Region

Another important thing we should mention here is a very intriguing fact regarding the breast cancer fusion gene, *ERBB2-GRB7*, which is a fusion between genes located in a close neighborhood in the human genome. Even though the fusion gene had been identified in breast cancer patients, the copy number amplicon (the one harboring the independent *ERBB2* and *GRB7* genes) had previously been reported in other cancer types, including gastric cancer [13,14]. In addition, the authors demonstrated that the co-amplification of those genes in the amplicons could result in simultaneous up-regulation in their expression and also might contribute to carcinogenesis in an orchestrated, cooperative, and synergistic manner for their oncogenic activities. 

**Figure 4:**
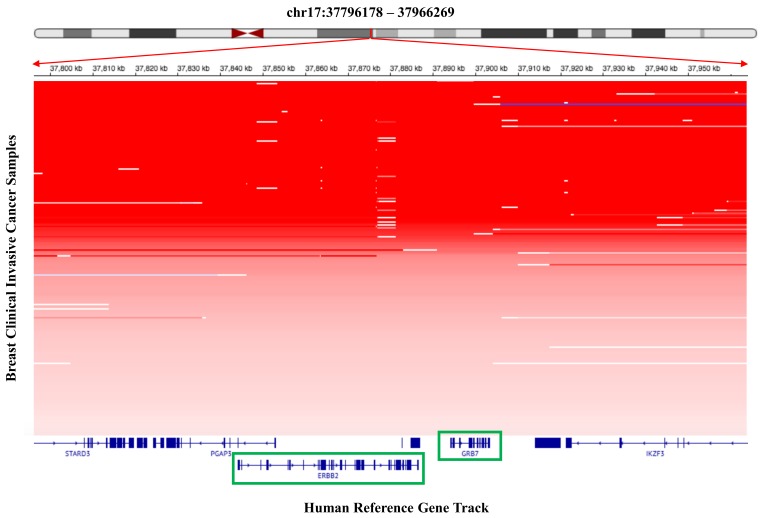
Co-amplification of *GRB7* within *ERBB2* genomic amplicon. The top panel shows *ERBB2* genomic amplicon region (indicated by a red color line) in a human chromosome ideogram. The two red arrowheads indicate an expanded *ERBB2* genomic amplicon region (chr17:37796178-37966269) harboring several co-amplified genes, which is scaled by a nucleotide position interval ruler for the human chromosome 17. The middle panel shows overall copy number amplifications of the *ERBB2* genomic amplicon involving several genes within the regions surrounding the *ERBB2* locus, including *GRB7*, in TCGA breast clinical invasive carcinoma samples. The denser the red color is, the higher the degree of the copy number amplification in the genomic region is. The bottom panel shows genomic exon-intron structures of those genes residing within the *ERBB2* amplicon.

**Figure 5:**
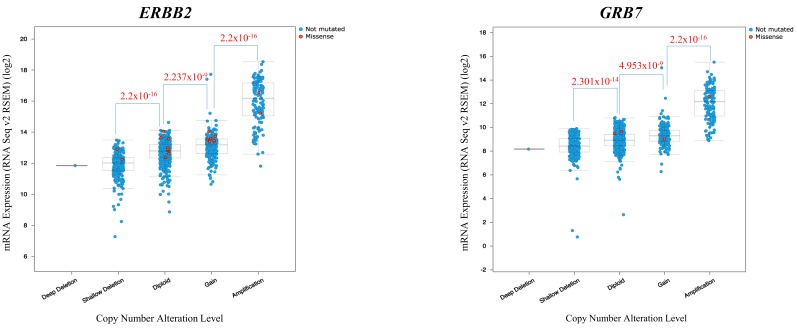
Relationships between copy number alteration and mRNA expression for *ERBB2* and *GRB7* in clinical breast invasive carcinoma samples. *p*-values were calculated by Wilcoxon rank sum test, except for only one breast carcinoma sample showing deep deletion of both *ERBB2* and *GRB7*.

In order to consider whether this might be a relatively generalized phenomenon in other cancer types, we analyzed the copy number amplification status within the close genomic neighborhood for the well-known oncogene, *ERBB2*, in TCGA clinical breast cancer samples, and also the relationship between copy number alteration and expression for the genes located within the genomic neighborhood. As shown in Figure 4, nearly 18% of clinical breast invasive carcinoma samples in TCGA database had shown high amplification of the genomic region that harbored *ERBB2*, *GRB7* and other neighboring genes. In addition, in those clinical breast invasive carcinoma samples with the amplified genomic region, there are statistically significant differences (*p* value calculations by Wilcoxon rank sum test) in mRNA expression level among different copy number alteration levels for *ERBB2* and *GRB7*, respectively (Figure 5). The higher their copy numbers are, the higher their mRNA expression levels are, which is statistically significant. This phenomenon implies that either fusion or co-amplification for those genes might be nearly equally suitable structures for their orchestrated and cooperative contribution to carcinogenesis. In the upcoming years, we may see more surprising results in considering how such cooperation between genes located within other recurrently prevalent oncogenic amplicons is and what kinds of structural bridging between them might be suitable for consequently promoting carcinogenesis.

## 9. Clinical and Therapeutic Implication of Fusion Genes in Breast Cancers

Even though numerous fusion genes, which are vastly diverse in terms of fusion gene type and fusion structure, are nowadays being rapidly identified in clinical breast cancer samples, validation of their involvement in carcinogenesis and the establishment for their clinical and therapeutic application as useful biomarkers require time-consuming, big money-consuming, painstaking and long-term processes. Nevertheless, in recent years, there has been some convincing progress in this field, especially regarding breast cancer. *ETV6-NTRK3*, which had been identified as a causative fusion oncogene in over 90% of secretary breast cancer samples, encodes a dimerization domain of the ETV6 transcription factor, conjoined to kinase domain of NTRK3 [15]. Such in-frame fusion enables a constitutive ligand-independent dimerization and auto-phosphorylation, consequently causing an oncogenic transformation of mammary epithelial breast cells [15]. Upon administration, larotrectinib (NTRK1/2/3 inhibitor, LOXO-101) binds to Trk (tropomyosin receptor kinase), causing the prevention of the neurotrophin-Trk interaction and Trk activation, and consequently resulting in both the induction of cellular apoptosis and the inhibition of cell growth in tumors overexpressing Trk [16]. At present, protein kinase inhibitors, such as NTRK1/2/3 inhibitor, LOXO-101, are now being tested for the therapeutic treatment of patients with solid tumors harboring the fusion *ETV6-NTRK3* in phase II clinical trials [16]. 

The recurrent conjoined gene *ESR1-CCDC170* occurring in nearly 4% of ER-positive clinical breast cancer patients has been proven to be a causative fusion oncogene underlying breast carcinogenesis [17]. Efforts for identifying inhibitors for therapeutic intervention of this oncogenic fusion are still under investigation. As mentioned in the previous section, it has been substantiated that the proliferation of breast cancer cells harboring the two conjoined genes, *SCNN1A-TNFRSF1A* and *CTSD-IFITM10*, could be decreased by targeting them with the corresponding siRNAs. This demonstrates the possibility that siRNA-based therapeutic drugs are suitable to be applied to the clinical treatment of cancers harboring a variety of other oncogenic fusion gene types in the future.

## 10. Conclusions

All the fusion gene types mentioned above in this paper and all the genomic regions harboring their translocation breakpoints and conjoining points should be chosen as priority targets for screening fusion gene biomarkers representing each of the diverse and extremely heterogeneous breast cancer subtypes in the coming years. For instance, the genomic regions across the translocation breakpoints or conjoining points generating the recurrent fusion genes mentioned in this paper should be chosen for designing forward or reverse primers for RT-PCR (reverse transcriptase-polymerase chain reaction) test and also selected as candidate regions for targeted RNA sequencing. With the advent of a next generation deep sequencing era guaranteed by a decreasing cost, the relationship between conjoined genes and carcinogenesis will become increasingly transparent. Also, the application of the CRISPR-CAS9 system to the high-throughput genome-wide functional validation of those fusion and conjoined genes will make our discovery process of breast cancer fusion gene biomarkers accelerate excitingly far beyond our present imagination.

## Figures and Tables

**Table 1:** Fusion Gene Transcripts involving Tumor Suppressor Genes in Breast Cancer.

Gene_A	Gene_B	TSG	A_chr	B_chr	Junction_A	Junction_B	Frame	FusionPair
*TSC2*	*TMEM204*	*TSC2*	16	16	2098754	1604783	Out-of-frame	*TSC2__TMEM204*
*ARID1A*	*THBS3*	*ARID1A*	1	1	27059283	155172928	In-frame	*ARID1A__THBS3*
*ARID1A*	*YTHDF2*	*ARID1A*	1	1	27059283	29064170	In-frame	*ARID1A__YTHDF2*
*AXIN1*	*ITFG3*	*AXIN1*	16	16	402369	299548	5UTR-5UTR	*AXIN1__ITFG3*
*AXIN1*	*ITCH*	*AXIN1*	16	20	396148	32957200	CDS-5UTR	*AXIN1__ITCH*
*AXIN1*	*IFT140*	*AXIN1*	16	16	354304	1576797	Out-of-frame	*AXIN1__IFT140*
*CASP8*	*ALS2CR12*	*CASP8*	2	2	202131514	202195556	In-frame	*CASP8__ALS2CR12*
*CDH1*	*CCDC132*	*CDH1*	16	7	68857529	92952923	In-frame	*CDH1__CCDC132*
*CDH1*	*NPFFR2*	*CDH1*	16	4	68772314	73003757	In-frame	*CDH1__NPFFR2*
*CDH1*	*DEXI*	*CDH1*	16	16	68842751	11023416	CDS-3UTR	*CDH1__DEXI*
*CCDC132*	*CDH1*	*CDH1*	7	16	92940584	68862077	In-frame	*CCDC132__CDH1*
*ASXL1*	*TM9SF4*	*ASXL1*	20	20	30956926	30720816	CDS-5UTR	*ASXL1__TM9SF4*
*ASXL1*	*PDRG1*	*ASXL1*	20	20	30959677	30536685	3UTR-CDS	*ASXL1__PDRG1*

**Table 2:** Fusion Gene Transcripts involving Chromatin Modifier Genes in Breast Cancer.

FusionPair	Gene_A	Gene_B	A_chr	B_chr	Junction_A	Junction_B	Frame
*KDM5A__ANO2*	*KDM5A*	*ANO2*	12	12	427272	5708795	In-frame
*CHD4__DTX1*	*CHD4*	*DTX1*	12	12	6690210	113515229	In-frame
*CTPS2__RBBP7*	*CTPS2*	*RBBP7*	X	X	16716359	16871965	In-frame
*USP21__PVRL4*	*USP21*	*PVRL4*	1	1	161134040	161042675	In-frame
*MANF__SETD2*	*MANF*	*SETD2*	3	3	51423766	47108608	In-frame
*XPC__RCOR1*	*XPC*	*RCOR1*	3	14	14209757	103177272	In-frame
*WNK1__KDM5A*	*WNK1*	*KDM5A*	12	12	939326	432947	In-frame
*NSD1__CDH23*	*NSD1*	*CDH23*	5	10	176563031	73434869	In-frame
*CDH23__NSD1*	*CDH23*	*NSD1*	10	5	73501678	176618885	In-frame
*SMARCA4__DNM2*	*SMARCA4*	*DNM2*	19	19	11136184	10930656	In-frame
*KDM5A__NINJ2*	*KDM5A*	*NINJ2*	12	12	404739	675344	In-frame
*MLL3__CLIP2*	*MLL3*	*CLIP2*	7	7	151871216	73811404	In-frame
*B4GALT7__NSD1*	*B4GALT7*	*NSD1*	5	5	177035623	176618885	In-frame
*MBD2__DOCK2*	*MBD2*	*DOCK2*	18	5	51715244	169502951	In-frame
*CREBBP__NUBP1*	*CREBBP*	*NUBP1*	16	16	3819175	10846467	In-frame
*ARHGEF17__EP300*	*ARHGEF17*	*EP300*	11	22	73022875	41545042	In-frame
*HDAC1__KPNA6*	*HDAC1*	*KPNA6*	1	1	32790154	32620189	In-frame
*TBC1D9__SMARCA5*	*TBC1D9*	*SMARCA5*	4	4	141560240	144438508	In-frame
*TET3__DGUOK*	*TET3*	*DGUOK*	2	2	74317174	74184252	In-frame
*USP22__MYO19*	*USP22*	*MYO19*	17	17	20945978	34852250	In-frame
*KDM4B__UHRF1*	*KDM4B*	*UHRF1*	19	19	5144913	4929234	In-frame
*MYSM1__APOM*	*MYSM1*	*APOM*	1	6	59160801	31624249	In-frame
*HDAC2__TRDN*	*HDAC2*	*TRDN*	6	6	114292021	123545276	In-frame
*ARID1A__THBS3*	*ARID1A*	*THBS3*	1	1	27059283	155172928	In-frame
*TET3__C2orf78*	*TET3*	*C2orf78*	2	2	74275538	74040604	In-frame
*NSD1__ZNF346*	*NSD1*	*ZNF346*	5	5	176619020	176489059	In-frame
*ARID1A__YTHDF2*	*ARID1A*	*YTHDF2*	1	1	27059283	29064170	In-frame
*SETDB1__PKP4*	*SETDB1*	*PKP4*	1	2	150915164	159477502	In-frame
*TRAF3__RCOR1*	*TRAF3*	*RCOR1*	14	14	103342862	103173688	In-frame
*KDM2A__RHOD*	*KDM2A*	*RHOD*	11	11	66888829	66833367	In-frame
*SCMH1__EIF2B3*	*SCMH1*	*EIF2B3*	1	1	41578955	45316675	In-frame
*CREBBP__ZNF500*	*CREBBP*	*ZNF500*	16	16	3929833	4803059	In-frame
